# Reasons for truly negative cytology reports preceding the diagnoses of invasive cervical cancer—Results of a false‐negative cytology audit in Polish Cervical Cancer Screening Programme

**DOI:** 10.1002/cam4.6024

**Published:** 2023-05-22

**Authors:** Anna Macios, Katarzyna Komerska, Andrzej Nowakowski

**Affiliations:** ^1^ Department of Cancer Prevention The Maria Sklodowska‐Curie National Research Institute of Oncology Warsaw Poland; ^2^ Doctoral School of Translational Medicine, Centre of Postgraduate Medical Education Warsaw Poland

**Keywords:** cervical cancer, cytology, false‐negative cytology audit, false‐negative result, screening

## Abstract

**Background:**

False‐negative (FN) results in cervical cancer (CC) screening pose significant risk for participants and should be audited. The aim of the study was to analyse the results of audit of FN slides collected in 2010–2013 in Polish Cervical Cancer Screening Program (CCSP) and to seek for risk factors of obtaining true‐negative result (TN; not containing abnormal cells as confirmed in audit) before CC diagnosis.

**Methods:**

Screening database was merged with National Cancer Registry to identify negative slides preceding histologically confirmed CC diagnosis up to 42 months. Two blinding slides were randomly assigned per each FN. The whole set was reassessed independently by three pathologists with 30 years of experience in cytology evaluation. Final audit result was established in the case of ≥2 coherent reports. Agreement rates and kappa (*κ*) coefficients were calculated. Logistic analysis of risk factors for obtaining TN result was performed.

**Results:**

Of 374 included FNs, 204 were considered abnormal (54.6%) and 91 were confirmed negative for intraepithelial neoplasia (24.3%). Agreement between experts was moderate for FNs (*κ* = 0.266) and fair for blinding slides (*κ* = 0.142) when grouping abnormal slides. Adenocarcinoma diagnosis elevated the risk of TN result (OR = 3.83); detection of macroscopic changes on the cervix and smoking lowered the risk (OR = 0.39, OR = 0.40 respectively).

**Conclusions:**

Misinterpretation was the main reason for FN cytology in the CCSP which indicated the need of further personnel training to increase screening quality. Rather low agreement between auditors requires further insight. A standardised process of auditors' selection should be planned to increase audit quality.

## 1 INTRODUCTION

Cervical cancer (CC) screening is an effective method to reduce CC incidence and mortality.^1^ However, sensitivity and reproducibility of cytology as a screening test may be limited^2^ and cause false‐negative (FN) results. Therefore, quality assurance on each step of the screening process, including evaluation of cytological slides, is an essential component of effective CC screening process along with the high coverage of the target population.^3^, ^4^ Since cytology is the most commonly used triage test in primary high‐risk human papillomavirus (HR‐HPV)‐based screening protocols, its quality will be even more important for correct identification of HPV‐positive women who have already developed intraepithelial lesions.

According to the *European Guidelines for quality assurance in cervical cancer screening*, audit of history of all CC cases should be performed, including re‐evaluation of false‐negative slides, that is, slides reported as negative for intraepithelial lesions and preceding CC diagnosis (called interval cancer, IC) in a given interval.^4^ FNs should be mixed with randomly selected control slides and reassessed in both blinded and unblinded manner. The most common reasons for the phenomenon of negative cytology reports before the CC diagnosis include misinterpretation of a slide as negative, inaccurate sampling and rapid cancer development. Correctly identified reasons of FNs may allow for implementation of corrective actions and improvement of screening programme effectiveness. In practice, the audit of FNs is indeed executed in many countries but with heterogeneous methodology.^5^

In Poland, the Cervical Cancer Screening Programme (CCSP) was commenced in late 2006 among insured women between 25 and 59 years old eligible for a cytology once in a 3‐year interval.^6^ Majority of screening tests has been conventional slides; liquid‐based cytologies (LBCs) constituted only a small fraction of them. In 2021, samples were collected by gynaecologists and midwives in over 1,500 clinics and evaluated in 74 laboratories countrywide. Around 60 colposcopy clinics performed further work‐up of positive screening results, that is, at least atypical squamous cells—cannot exclude high‐grade squamous intraepithelial lesions (ASC‐H) or more severe diagnosis or repeated low‐grade squamous intraepithelial lesions (LSIL) or atypical squamous cells of undetermined significance (ASC‐US) reported accordingly to the modified Bethesda system.^6^, ^7^, ^8^ HPV testing is not reimbursed by the National Health Fund within screening programme and no information on HPV status could therefore be input in triage protocol. After introduction of CCSP in Poland, an acceleration in the decline in both CC incidence and mortality trends was noticed.^9^ Although coverage of the programme has never exceeded 25%, CC prevention became popular and combined coverage of the programme and opportunistic screening rose by over 20% in some age groups between 2004 and 2009.^7^, ^10^, ^11^

More intensive actions towards quality monitoring in Polish CCSP have been undertaken since 2018 when first FN audit was performed, covering women screened in 2010–2011 in one of Polish regions. The results of experts' evaluation indicated errors in initial evaluation as a reason for almost half of FN reports. In only 10% cases, the screening result was confirmed as NILM and 6% slides were considered unsatisfactory for evaluation. The pilot results suggested the need for more in‐depth investigation of assessment quality and corrective actions.^12^

The aim of this study was to analyse the results of FN audit which took place in 2019–2021 in Poland and to seek for reasons of FNs occurrence as well as for potential risk factors of obtaining truly negative cytology results preceding diagnosis of ICs, defined as FNs confirmed as no intraepithelial lesions or malignancy (NILM) by experts' evaluation. Auditors' concordance will also be discussed.

## 2 MATERIALS AND METHODS

CCSP data is collected in the IT System for Prevention Monitoring (pol. System Informatyczny Monitorowania Profilaktyki, SIMP). Clinical information on macroscopic assessment of the cervix, detailed results of the laboratory evaluation and results of additional diagnostic procedures performed in case of abnormal screening result is gathered in SIMP for each participant.

The questionnaire filled by each woman before the sampling consisted of questions regarding, among others, (1) education level, (2) number of deliveries, (3) smoking status, use of: (4) oral contraceptives, (5) hormonal replacement therapy, (6) intrauterine device; (7) elevated risk of CC occurrence (due to HPV infection in the past, human immunodeficiency virus (HIV) infection or immunosuppressive drugs intake). Clinician collecting the smear evaluated the cervix and reported on the presence of: (1) colpovaginitis, (2) ectropion, (3) papilloma, (4) cervical distortion, (5) overgrowth, (6) necrosis, (7) polyp, (8) tumour, (9) infiltration and (10) ulceration. Questionnaire was sent to the laboratory along the slide and was given to the cytodiagnostician. Results of Pap smear evaluation coded according to the modified Bethesda system contained detailed information on the presence of each of the following microorganisms on the slide: *Trichomonas vaginalis*, *Candida albicans*, *Herpes simplex viruses*, *Bacterial vaginosis*, *Actinomyces*, *Chlamydia trachomatis* or other unspecific bacterial infection and changes in bacterial flora. In case of further diagnostics executed within the CCSP, the result of colposcopic examination and histological report was also entered into the SIMP.

Each case of cancer diagnosed in Poland is required by law to be reported in the Polish National Cancer Registry (NCR). Cancer notification include, among others, date of diagnosis, codes of diagnosis—according to the *International Statistical Classification of Diseases and Related Health Problems, 10th revision* ^13^ (ICD‐10)—and histological type of diagnosed cancer—according to the *International Classification of Diseases for Oncology, 3rd revision*.^14^ Based on this information, three histological types of CC cases were distinguished: (1) squamous cell carcinoma (SCC); (2) adenocarcinoma (ADC); (3) other rare types of carcinoma (OTC). Specific histotypes included in each group can be found in the Appendix S1.

The SIMP database with information on all CCSP participants in 2010–2013 was merged with NCR by personal identification numbers. Records with administrative errors were excluded (screening after the CC diagnosis; screening after the reported date of death; diagnosis after the reported date of death). Normal (NILM) and abnormal (ASC‐US or more severe results) screening slides preceding CC diagnosis in 42 months were identified. All slides with negative results were considered potential FNs and were qualified for the audit. Slides with positive results were considered true positives (TPs). Participants with *unsatisfactory for evaluation* results were rejected from analysis.

FN slides were afterwards audited according to the *European Guidelines* ^4^: they were blinded and mixed with randomly chosen screening slides. Two additional smears were drawn from all CCSP screens evaluated by the specific laboratory in 2010–2015 per each FN. Selected slides were requested from labs but a part of slides was not provided for various reasons: Some laboratories had already destroyed their NILM slides which was in line with legal regulations, and one laboratory declined sending slides doubting in legal basis of our request.

Reassessment was performed independently by three experts—professors in the field of pathology with over 30 years of experience in Pap smear evaluation. They were personally chosen by the Head of Central Coordinating Centre for Cervical Cancer Screening as three most‐valued and best‐experienced pathologists in the country. Experts were aware of subject of the study but not of the composition of slides set. They were also not provided with the woman's questionnaire filled before sampling. Results were reported by experts according to Bethesda system and subsequently coded according to two following types of coding: (1) general: unsatisfactory for evaluation/NILM/abnormal (ASC‐US or more severe lesions); (2) aggregated: unsatisfactory for evaluation/NILM/low‐grade lesions (ASC‐US, LSIL)/high‐grade lesions (ASC‐H, high‐grade squamous intraepithelial lesions (HSIL), SCC, atypical glandular cells (AGC), ADC). Final result of audit was established in each type of coding in case of agreement of majority of experts (at least two out of three pathologists); subsequently, for some slides with no experts' agreement, no final diagnosis could have been stated. We decided to merge specific Bethesda diagnoses into broader categories, such as low‐grade and high‐grade lesions, because these categories trigger similar triage examinations. Also due to independence of experts' diagnoses and well‐known subjectivity of cytology evaluation, this procedure ensured the possibility of establishing final diagnosis for most of slides.

A group of FN slides confirmed in the audit as NILM by at least two experts was considered as truly negative (TN) smears preceding cancer diagnosis. To seek potential risk factors of such a report, TNs group was compared to the TPs group. Only diagnoses with histologically confirmed invasion were included in the study. Retrieving histological blocks for reevaluation and reconfirmation was not possible and final histological diagnosis was based on morphological codes reported in NCR according to the ICD‐0‐3. List of potential risk factors was completed according to the literature and expert knowledge and was adjusted for availability of data. Final risk factors list included: age at screen, smoking status, any parturitions given, use of hormones (any of: oral contraceptives, hormone replacement therapy or intrauterine device), any macroscopic changes on the cervix, any microorganisms reported on the slide and histological type of diagnosed CC.

### 2.1 Statistical analysis

Descriptive statistics were used to depict important features of slides included in the study: median with interquartile range for non‐normally distributed continuous variables and numbers with percentages for qualitative variables. Appropriate statistical tests were performed to check significance of differences, including U Mann–Whitney test, chi‐squared test, Fisher's exact test. Normality was checked with Shapiro–Wilk test.

For each type of coding kappa coefficient (*κ*) was computed to assess agreement achieved by experts beyond the chance. Confidence intervals (CIs) for kappa coefficient were calculated using bootstrap method with 1,000 replications. Rates of concordant diagnoses were also calculated with corresponding CI.

The multivariable logistic regression analysis of factors potentially influencing the risk of obtaining true‐negative (TN) result preceding invasive cervical cancer diagnosis within 42 months was performed using the stepwise method of variables selection with *p* < 0.1 considered significant to construct the final model. Univariate analyses were also conducted.

All tests were two‐sided and *p*‐value <0.05 was established as indicating statistically significant differences. Analyses were performed in the Stata 15 software.^15^

## 3 RESULTS

### 3.1 CCSP in Poland in 2010–2013 and FNs identification

**FIGURE 1:**
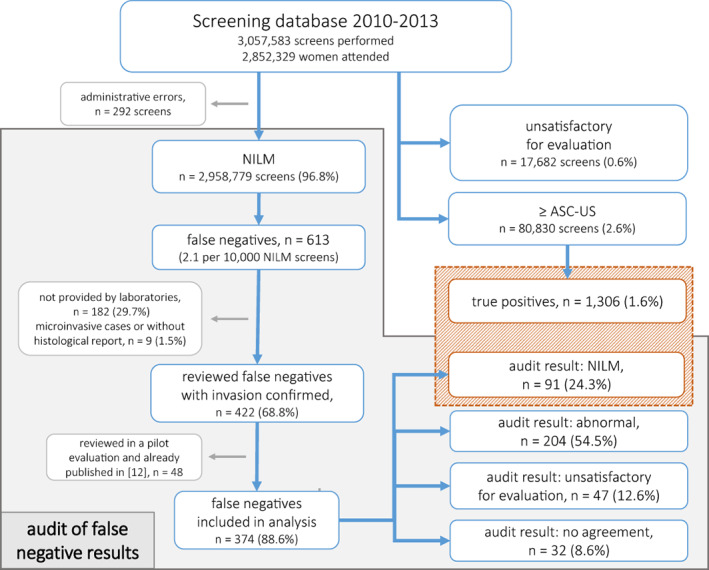
Study flowchart.

In 2010–2013 there were 3,057,583 screening Pap smears performed in Poland, including 80,830 abnormal (2.6%) and 17,682 unsatisfactory for evaluation slides (0.6%). Among screening tests, 613 FNs (2.1 per 10,000 NILM screens) and 1,306 TPs (1.6% of all screens) were identified (see Figure 1). Additional 1,226 slides were randomly selected to blind experts' evaluation.

Audit was performed in 2018–2021. Laboratories refused or were unable to provide a part of slides and overall 431 FNs and 960 blinding slides were handed over and reassessed by all three expert pathologists. Due to unconfirmed cancer invasion status, nine cases were excluded from analysis as well as subsequent 48 FN and 94 blinding slides acquired in 2018 and reassessed by different experts as these results were published elsewhere.^12^ Overall, 374 potential FNs and 866 blinding slides were left (61.0% and 70.6%, respectively). As shown in Table 1, FN slides included in analysis and those excluded or not provided by laboratories did not differ significantly except from report on microorganism infection on the cytological slide (*p* = 0.028). We therefore assumed that conclusions drawn from the analysed set were generalisable to all identified FN slides.

**TABLE 1:** Comparison of potential false‐negative (FN) slides: (1) re‐evaluated within the FN audit with histological confirmed invasion and included in analysis and (2) identified as FN but not provided by laboratories or excluded from analysis due to the uncertain invasion status or previously analysed by Komerska et al.^12^

Risk factors of true‐negative cytology result preceding diagnosis of interval cervical cancer	FN re‐evaluated within audit and analysed (1), *n* = 374	FN not provided by laboratory or excluded from analysis (2), *n* = 239	*p*‐value
Age at screen, median (IQR)	48 (17)	49 (16)	0.621*
Smoking, *n* (%)			0.173**
No	235 (62.8)	134 (56.1)	
Yes	115 (30.7)	91 (38.1)	
Quit	24 (6.4)	14 (5.9)	
Any delivery in the past, *n* (%)	339 (90.6)	217 (90.8)	0.949**
Use of hormone replacement therapy, oral contraceptives or intrauterine device, *n* (%)	46 (12.3)	34 (14.2)	0.490**
Macroscopic changes on the cervix, *n* (%)	93 (24.9)	66 (27.6)	0.449**
Microorganism infection on the cytological slide, *n* (%)	137 (36.6)	67 (28.0)	0.028**
Recommendations on rescreening after anti‐inflammatory treatment, *n (%)*	41 (11.0)	19 (7.9)	0.221**
Recommendations on rescreening after Meigs' test, *n (%)*	6 (1.6)	4 (1.7)	1.000***
Histological type of diagnosed cervical cancer, *n* (%)			0.506**
Squamous‐cell carcinoma	273 (73.0)	175 (73.2)	
Adenocarcinoma	82 (21.9)	41 (17.2)	
Other types of carcinoma	19 (5.1)	11 (4.6)	

* *U* Mann–Whitney test

** Chi‐squared test

*** Exact Fisher's test.

### 3.2 Audit results

#### 3.2.1 FN slides

Of 374 FN slides, 47 were evaluated as unsatisfactory for evaluation (12.6%), 91 as normal (24.3%) and 204 as abnormal (54.6%), including 15 low‐grade (4.0%), 159 high‐grade lesions (42.5%) and 30 cases with no agreement on lesion severity (8.0%). No final diagnosis was made for 32 FN slides (8.6%; Table 2).

**TABLE 2:** Results of false‐negative slides audit performed in 2019–2021 in Polish Cervical Cancer Screening Programme.

Final evaluation	False‐negative slides			Additional blinding slides by screening result			
	Agreed by 2 or 3 experts	Agreed by 2 experts	Agreed by 3 experts	Unsatisfactory for evaluation	Normal	Abnormal	Overall
No agreement	32 (8.6)			1 (11.1)	53 (6.4)	0 (0.0)	54 (6.2)
Unsatisfactory for evaluation	47 (12.6)	36 (9.6)	11 (2.9)	5 (55.6)	115 (13.9)	1 (3.7)	121 (14.0)
Normal	91 (24.3)	78 (20.9)	13 (3.5)	2 (22.2)	464 (55.9)	2 (7.4)	468 (54.0)
Abnormal	204 (54.6)	77 (20.6)	127 (34)	1 (11.1)	198 (23.9)	24 (88.9)	223 (25.8)
Low‐grade lesions*	15 (4.0)	15 (4.0)	0 (0)	0 (0.0)	42 (5.1)	3 (11.1)	45 (5.2)
High‐grade lesions**	159 (42.5)	70 (18.7)	89 (23.8)	0 (0.0)	102 (12.3)	15 (55.6)	117 (13.5)
No agreement on lesions' severity	30 (8.0)			1 (11.1)	54 (6.5)	6 (22.2)	61 (7.0)
Total no of re‐evaluated slides	374			9	830	27	866

*Note*: Final evaluation was established in case of agreement of at least two of three experts involved in the audit.

* Low‐grade lesions included: ASC‐US, LSIL

** High‐grade lesions included: ASC‐H, HSIL, AGC, AIS, SCC, ADC.

In general coding, exactly two experts agreed on final diagnosis in 191 cases (51.1%) and all three experts agreed in 151 cases (40.4%). In aggregated coding at least two coherent experts' diagnoses were stated in 312 cases (83.4%): exactly two experts agreed in 199 cases (53.2%) and all three experts in 113 cases (30.2%). High‐grade lesions were reported by at least two experts in 159 slides (42.5%) and by all experts in 89 slides (23.8%).

Additional analyses with age stratification can be found in the Appendix S1.

#### 3.2.2 Blinding slides

The majority of blinding slides were primarily assessed as negative (*n* = 830, 95.8%) with little number of abnormal (*n* = 27, 3.1%) and unsatisfactory for evaluation slides (*n* = 9, 1.0%). Among slides classified as normal in screening, in 464 cases (55.9%) this report was confirmed by experts, and in 198 slides (23.9%) screening result was underestimated. Also 115 screens were reclassified as unsatisfactory for evaluation (13.9%). Among primarily abnormal slides, 24 were approved as truly abnormal (88.9%). All abnormal results gained the agreement of at least two auditors and, therefore, final evaluation. Slides assessed as unsatisfactory for evaluation in screening were mainly confirmed by experts' diagnosis (5/9, 55.6%) but sample size impeded strict conclusions.

### 3.3 Auditors performance

In general, coding percentage of agreement between each pair of experts was lower for additional slides (36.6%–62.0%) than for FNs (51.3%–64.4%) and so was the kappa coefficient. According to Landis and Koch scale,^16^ in general coding there was fair to moderate agreement between pairs of auditors for FNs (*κ* ranging from 0.201 to 0.387, overall 0.266) and only slight to fair agreement for blinding slides (*κ* from 0.149 to 0.248, overall 0.142). In aggregated coding agreement was lower due to the higher number of possible choices. Rate of agreement did not exceed 60% in group of both blinding and FN slides and overall kappa coefficient represented only slight agreement for additional slides (*κ* = 0.129) and moderate for FNs (*κ* = 0.236; Table 3).

**TABLE 3:** Agreement between three experts (A, B, C) in independent evaluation of false‐negative and additional blinding slides during the audit of false‐negative cytological results in Polish Cervical Cancer Screening Programme in terms of rate of agreement and kappa coefficient with 95% CI.

Type of coding	Re‐evaluated slides	Agreement between experts, % (95% CI)			Kappa coefficient of agreement between experts, *κ* (95% CI)			
		A versus B	A versus C	B versus C	A versus B	A versus C	B versus C	Overall
General	False‐negatives	51.3 (46.3–56.4)	56.4 (51.3–61.4)	64.4 (59.4–69.1)	0.201 (0.135–0.270)	0.265 (0.199–0.340)	0.387 (0.308–0.463)	0.266 (0.214–0.321)
	Additional	43.0 (39.7–46.3)	36.6 (33.5–39.9)	62.0 (58.7–65.2)	0.201 (0.159–0.240)	0.149 (0.116–0.184)	0.248 (0.191–0.306)	0.142 (0.108–0.178)
Aggregated	False‐negatives	39.0 (34.2–44.1)	49.2 (44.1–54.3)	55.6 (50.5–60.6)	0.174 (0.122–0.230)	0.261 (0.206–0.325)	0.333 (0.268–0.396)	0.236 (0.193–0.284)
	Additional	35.3 (32.2–38.6)	33.3 (30.2–36.5)	59.2 (55.9–62.5)	0.167 (0.132–0.204)	0.145 (0.112–0.178)	0.220 (0.167–0.274)	0.129 (0.095–0.159)

### 3.4 Risk factors of obtaining true‐negative result before CC diagnosis

During the audit, 91 of potential FN slides were coherently assessed by at least two experts as negative for intraepithelial lesions. Among women screened in 2010–2013 with ASC‐US or more severe result, 1,306 were subsequently diagnosed with invasive CC within at most 42 months. Groups of potential FNs confirmed as negative in audit (henceforth called true negatives, TN) and TP were subsequently compared by the use of logistic regression modelling (Table 4).

**TABLE 4:** Results of logistic regression analysis of potential risk factors of true‐negative result preceding diagnosis of interval cervical cancer in Polish Cervical Cancer Screening Programme.

Risk factors of true‐negative cytology result preceding diagnosis of interval cervical cancer	True negatives preceding diagnosis of interval cervical cancer, *n* = 91	True positives preceding diagnosis of invasive cervical cancer, *n* = 1306	Multivariable logistic regression				Univariable logistic regression			
			OR	95% CI		*p*‐value	OR	95% CI		*p*‐value
Age at screen, median (IQR)	50 (16)	48 (14)					1.00	0.98	1.02	0.963
Smoking, *n* (%)										
No	61 (67.0)	608 (46.6)	1.00				1.00			
Yes	23 (25.3)	614 (47.0)	0.40	0.24	0.67	<0.001	0.37	0.23	0.61	<0.001
Quit	7 (7.7)	84 (6.4)	0.87	0.38	2.01	0.751	0.83	0.37	1.88	0.655
Any parturitions in the past, *n* (%)	82 (90.1)	1203 (92.1)					0.78	0.38	1.60	0.497
Use of hormone replacement therapy, oral contraceptives or intrauterine device, *n* (%)	12 (13.2)	129 (9.9)					1.39	0.74	2.61	0.313
Macroscopic changes on the cervix^a^, *n* (%)	17 (18.7)	520 (39.8)	0.39	0.22	0.67	0.001	0.35	0.20	0.60	<0.001
Microorganism infection on the cytological slide^b^, *n* (%)	23 (25.3)	243 (18.6)	1.66	0.99	2.77	0.053	1.48	0.90	2.42	0.119
Histological type of diagnosed cervical cancer, *n* (%)										
Squamous‐cell carcinoma	65 (71.4)	1165 (89.2)	1.00				1.00			
Adenocarcinoma	22 (24.2)	96 (7.4)	3.83	2.22	6.59	<0.001	4.11	2.43	6.95	<0.001
Other types of carcinoma	4 (4.4)	45 (3.4)	1.81	0.62	5.27	0.280	1.59	0.56	4.56	0.386

^a^ Include: colpo‐vaginitis, ectropion, papilloma, distortion, overgrowth, necrosis, polyp, tumour, infiltration, ulceration.

^b^ Include: *Trichomonas vaginalis, Candida albicans, Herpes simplex viruses, Bacterial vaginosis, Actinomyces, Chlamydia trachomatis* or other unspecific bacterial infection and changes in bacterial flora.

In the stepwise procedure of variables selection age at screen, parity status and hormone use were excluded. Results of final model showed 3.83 times higher odds of obtaining TN cytology for women subsequently diagnosed with ADC compared to those with SCC (OR = 3.83, 95% CI 2.22–6.59). Odds were lower for smoking women (OR = 0.40, 95% CI 0.24–0.67) and for those with macroscopic changes on the cervix (OR = 0.39, 95% CI 0.22–0.67). Borderline significance was indicated for signs of microorganisms infection on the slide (OR = 1.66, 95% CI 0.99–2.77, p = 0.053). Estimates of multivariable model were confirmed by the univariate regression.

## 4 DISCUSSION

First FN audit in Poland performed according to *European guidelines* ^4^ pointed out misclassification as a main reason for FN occurrence in CCSP: in 54.6% of slides experts agreed on the presence of abnormal cells, and in 42.5%, they agreed on high‐grade lesions existence. Agreement between experts was insufficient since it was interpreted as slight to moderate only. Developed logistic regression model indicated ADC diagnosis as a factor increasing the risk of obtaining TN result before CC diagnosis and both smoking cigarettes and macroscopically visible changes in the cervix were shown to decrease the risk.

### 4.1 Results of FN audit

Interval cervical cancer audit should be annually performed in each screening programme,^4^ but no coherent methodology has been adapted in this activity worldwide yet. According to the paper by Fitzpatrick et al., distinct audit approaches have been implemented in countries with ongoing CC screening programmes.^5^ Differences have been related to various fields: blinding experts to cancer status of women by using additional slides mixed with FNs for re‐evaluation as well as gaining women's consent for participating in audit and informing them on results. Audit should be performed in an unified manner to make results in different countries comparable.

As the worldwide screening has been gradually switching from cytology to primary HR‐HPV testing, the number of FN cases will probably decrease, however, it would not reach an absolute zero.^17^ Still, FN cytology audit would probably remain an essential part of quality assurance in CC screening after switch to primary HPV‐based screening since cytology would still be a part of all triage protocols despite the use of HPV16/18, extended or complete genotyping. In case of HPV‐positive result, ensuring lowest possible FN level would allow proper and timely diagnosis for at‐risk women developing cervical lesions.

Results of FN audit in Poland showed over 50% reclassification of normal slides in screening to abnormal ones. This rate seemed higher than in the UK audit of screening NILMs originated in 2013–2016, where the rate of slides upgraded to abnormal in unblinded evaluation was 41.1%–45.9%, depending on the age group.^18^ Even lower upgrading rate was reported in UK when auditing years of 2007–2010 (36.8%).^19^ In Ireland, among 196 women diagnosed with CC in 2008–2018 after NILM result, 88 screening reports were considered downgraded (44.9%).^20^ Since the auditors were unblinded in both UK and Ireland and were blinded in Poland, one might have expected lower rate of reclassification to abnormal slides in Poland; however, the situation was the opposite. On the other hand, older reports published in '90s were more consistent with our results and suggested about 52%–53% of reclassification of normal slides to abnormal.^21^, ^22^ This indicated that the quality of assessment screening cytology in Poland was suboptimal and needed improvement. The highest quality of cytology evaluation may be achieved by ongoing regular training and certification of staff involved in slides evaluation, as proposed by some studies^23^; other researchers suggested that only the level of slides' difficulty influences the agreement rates rather than staff's academic or professional degrees.^24^, ^25^

The occurrence of 24.3% of truly normal slides sampled before CC diagnosis might have resulted from: (1) location of lesions deep in endocervical canal hampering appropriate sampling; (2) improper sampling and (3) true lack of lesions at the moment of sampling and their rapid development and progression.

Slides reclassified as unsatisfactory for evaluation (12.6%) suggested improper sampling. High variations in rate of slides re‐evaluated as unsatisfactory for evaluation were reported in literature: from 0% to 81%.^8^, ^20^, ^26^, ^27^

### 4.2 Auditors' performance

Cytology is perceived as a subjective examination with limited reproducibility^28^ in case of CC screening. This phenomenon seemed to be confirmed by results of expert reevaluation in our audit. Expert auditors' agreement was lower than expected, with overall kappa of 0.266 and 0.142 in the general coding for FNs and for blinding slides, respectively. Expectation bias might have played a significant role during the auditing process. As experts were aware of the purpose of study, they might have expected an excessive number of FN slides. Also, as showed by Larson et al., some readers after abnormal interpretation of one slide are more likely to lowering the threshold for such an interpretation on subsequent ones.^29^ This might partially explain the high rate of abnormal results among the control slides used as a background, however, not the high discrepancies between auditors.

According to literature, narrow reproducibility of cervical cytology seemed not to depend on the reader's experience or scientific degree but on the difficulty level of the slides set.^24^, ^25^ Assuming high level of professionalism and skills of experts, we might hypothesise that selected slides were quite demanding and therefore posed significant problem for proper evaluation. Auditors enjoy the respect of screening community. However, we decided to verify whether the level of concordance between them resulted from the process of their selection. Experts for the next round of FN audit were chosen among those with best results in examination on a standardised set of slides.

### 4.3 Risk factors of obtaining true‐negative result preceding CC diagnosis

Analysis of risk factors for gaining TN cytology result before CC diagnosis is in line with the previous one where all FN slides have been included irrespective of audit results.^30^ In this study, macroscopic changes on the cervix and current smoking status were shown to decrease the risk of TN cytological report before CC diagnosis and the diagnosis of ADC increased the odds compared to SCC.

In CCSP, each diagnostician is provided with a report on any changes visible on the cervix during speculum examination. Therefore, it is possible that diagnosticians might have paid more attention to slides with any macroscopic changes reported which resulted in more accurate evaluation or, on the other hand, the result may have been potentially upgraded due to expectation bias. Occurrence of changes on the cervix may indicate disease process and it seems to be the main reason of lowering the risk of negative result before the CC diagnosis in women with positive macroscopic changes report.

The issue of lower performance of cervical cytology in case of ADC is well investigated in the literature.^31^, ^32^ Glandular lesions are typically located upper in the endocervical canal which hampers proper sampling and may lead to a TN result of cytology. Our analysis clearly confirmed the thesis of lower sensitivity of cytology for ADC detection compared to SCC with almost four times higher risk of TN result preceding CC diagnosis compared to TP ones. Important indication of almost two times higher risk of TN result obtaining in OTC group compared to SCC cases should be proved in a study with a higher number of participants.

Lower risk of TN result in smokers compared to non‐smokers may be related to the report showing that smoking increases the risk of SCC and does not influence the risk of ADC on the cervix.^33^, ^34^ As discussed in previous paragraph, sampling is more difficult and more TN may be expected in case of ADC which causes the confusing conviction of protective effect of smoking on obtaining TN results before CC diagnosis.

To conclude, the first audit of FN slides in Poland performed in a blinded manner by three experts showed over 50% underestimation of screening reports which might have indicated the need for improvement of diagnosticians' skills by additional training. ADC report was found as the factor inflating the risk of TN cytological report before CC diagnosis; smoking and macroscopic changes on the cervix reduced the risk. The reasons for limited concordance between auditors may cover: truly difficult for evaluation set of slides and varying experts' performance. In order to enhance concordance between the auditors and possibly the quality of audit in 2022, new experts were chosen based on the best results obtained in an examination process.

## AUTHOR CONTRIBUTIONS

**Anna Macios:** Data curation (equal); formal analysis (equal); investigation (equal); methodology (equal); writing – original draft (equal). **Katarzyna Komerska:** Data curation (equal); investigation (equal); methodology (equal); project administration (equal); writing – review and editing (equal). **Andrzej Nowakowski:** Conceptualization (equal); investigation (equal); methodology (equal); supervision (equal); writing – review and editing (equal).

## FUNDING INFORMATION

The study was financed by the Polish Ministry of Health through the National Cancer Control Programme within the objective of coordination and monitoring of quality of cervical and breast cancer screening.

## CONFLICT OF INTEREST STATEMENT

Authors declare no conflict of interests relevant to this article.

## ETHICS STATEMENT

The study was approved by the ethics committee of the Centre of Postgraduate Medical Education (110/2021).

## Supporting information


Appendix S1

## Data Availability

The data that support the findings of this study are available on reasonable request from the corresponding author.
