# Supplementary material for: Reasons for truly negative cytology reports preceding the diagnoses of invasive cervical cancer—Results of a false‐negative cytology audit in Polish Cervical Cancer Screening Programme
Source: Cancer Med. 2023 May 22;12(12):13800–10. doi: 10.1002/cam4.6024 (PMC10315736; doi:10.1002/cam4.6024)
Supplement: Supplementary file 1 — Appendix S1 [file CAM4-12-13800-s001.docx]

**Supplementary file 1**

**Histotypes included in specific histological subgroups**

(1) squamous cell carcinomas – including: papillary carcinoma, not otherwise specified (NOS); papillary squamous cell carcinoma; squamous cell carcinoma, NOS; squamous cell carcinoma, keratinizing, NOS; squamous cell carcinoma, large cell, nonkeratinizing, NOS; squamous cell carcinoma, small cell, nonkeratinizing; basaloid squamous cell carcinoma; verrucous carcinoma, NOS; squamous cell carcinoma in situ, NOS;

(2) adenocarcinomas – including: adenoma, NOS; ADC, NOS; papillary ADC, NOS; clear cell ADC, NOS; mixed cell ADC; endometrioid ADC, NOS; ADC, endocervical type; serous cystadenocarcinoma, NOS; mucinous ADC; mucinous ADC, endocervical type; adenosquamous carcinoma; adenoid basal carcinoma;

(3) other rare types of carcinoma – including: neoplasm, malignant; carcinoma, NOS; large cell neuroendocrine carcinoma; carcinoma undifferentiated, NOS; carcinoma anaplastic, NOS; combined small cell carcinoma; neuroendocrine carcinoma, NOS; mucoepidermoid carcinoma; leiomyosarcoma, NOS; adenosarcoma; carcinosarcoma, NOS; solid carcinoma, NOS.

**Results of false-negative audit stratified by age-group**

We analysed results of false negative (FN) audit according to age of women at the date of sampling. In the group of 25-29 year olds there were only 26 FN cases and therefore we excluded them from analysis. The lowest rate of FN slides confirmed by experts as NILM was observed in the age group of 30-39 year olds (17.6%). The pointwise estimations suggested the raising trend among 30-39, 40-49, 50-59 years old groups, with the highest of 28.7% in the oldest group. However, the raise was not statistically significant (Cochran-Armitage p for trend 0.472).

Supplementary Table 1. The results of false negative slides review stratified by women’s age. Women were divided in 10-years groups according to their age at sampling. *low grade lesions included: ASC-US, LSIL; ** high-grade lesions included: ASC-H, HSIL, AGC, AIS, SCC, ADC.

| **Final evaluation  of false-negative slides** | **age group** | | | | **overall** |
| --- | --- | --- | --- | --- | --- |
|  | **25-29** | **30-39** | **40-49** | **50-59** |  |
| **no agreement** | 1 (3.8) | 5 (5.9) | 14 (14.6) | 12 (7.2) | 32 (8.6) |
| **unsatisfactory for evaluation** | 2 (7.7) | 12 (14.1) | 14 (14.6) | 19 (11.4) | 47 (12.6) |
| **normal** | 8 (30.8) | 15 (17.6) | 20 (20.8) | 48 (28.7) | 91 (24.3) |
| **abnormal** | 15 (57.7) | 53 (62.4) | 48 (50) | 88 (52.7) | 204 (54.5) |
| **low-grade lesions*** | 1 (3.8) | 5 (5.9) | 5 (5.2) | 4 (2.4) | 15 (4) |
| **high-grade lesions**** | 13 (50) | 40 (47.1) | 33 (34.4) | 73 (43.7) | 159 (42.5) |
| **no agreement on lesions' severity** | 1 (3.8) | 8 (9.4) | 10 (10.4) | 11 (6.6) | 30 (8) |
| **Total no of re-evaluated slides** | 26 (100) | 85 (100) | 96 (100) | 167 (100) | 374 (100) |
